# Comparative effects of short- and long-distance sprint training on speed, change-of-direction ability, and intermittent aerobic endurance in collegiate basketball players

**DOI:** 10.3389/fphys.2026.1911961

**Published:** 2026-09-09

**Authors:** Yang Zhang, Jiayi Wang, Zhihai Wang

**Affiliations:** 1 China Basketball College, Beijing Sport University, Beijing, China; 2 College of Physical Education, Anhui Normal University, Wuhu, China

**Keywords:** acceleration, neuromuscular adaptation, strength and conditioning, team sport, training specificity

## Abstract

**Background:**

This study compared the effects of short-distance sprint training (SST) and long-distance sprint training (LST), under matched total training volume, on sprint performance, change-of-direction (COD) performance and intermittent aerobic endurance in collegiate basketball players.

**Methods:**

Thirty male collegiate basketball players were stratified by playing position before being randomly assigned to either the SST group (n = 15) or the LST group (n = 15). Both groups completed a 6-week sprint training intervention twice weekly. Before and after the intervention assessments included sprint performance (5 m, 10 m, 20 m, and 30 m sprint tests), COD performance (505 test, T-test, and COD deficit (CODD)), and intermittent aerobic endurance (Yo-Yo Intermittent Recovery Test Level 1 (Yo-Yo IR1)). Rating of perceived exertion (RPE) was monitored throughout the intervention. Data were analyzed using one-way analysis of variance and two-way repeated-measures ANOVA.

**Results:**

A significant group × time interaction observed only for 5 m sprint performance (*F* = 7.99, *p* = 0.022, η²_p_ = 0.222). No significant interaction effects or group main effects were observed for the remaining sprint, COD, intermittent aerobic endurance, or RPE variables (*p* > 0.05). However, significant time main effects were found for 10 m, 20 m, and 30 m sprint performance, 505 test, T-test, CODD, and Yo-Yo IR1 (*p* < 0.05), indicating that both training protocols improved these outcomes over time. Although the SST group showed descriptively greater percentage improvements in 5 m and 10 m sprint performance, and the LST group demonstrated larger percentage improvements in 20 m and 30 m sprint performance, COD performance, and Yo-Yo IR1, these differences were not statistically significant. RPE responses were similar between groups throughout the intervention.

**Conclusions:**

Under matched training volume conditions, both SST and LST effectively improved sprint, COD, and intermittent aerobic endurance performance after 6 weeks in male collegiate basketball players. SST elicited significantly greater improvements only in 5 m sprint performance, whereas both training protocols produced comparable improvements in the remaining outcomes. Our findings suggest that sprint distance is a meaningful programming variable that may shape basketball-specific physical adaptations.

**Clinical trial registration:**

https://www.chictr.org.cn, identifier ChiCTR2600122073.

## Introduction

Basketball is a prototypical high-intensity intermittent team sport, characterized by the recurrent alternation between brief bouts of maximal or near-maximal effort and periods of lower-intensity recovery (García et al., 2020). During competition, players are repeatedly required to execute accelerations, decelerations, sprints, and changes of direction (COD), with high-intensity accelerations (≥ 2 m·s^-^²), decelerations (≤ −2 m·s^-^²), and short-distance high-speed sprints constituting some of the most frequently observed movement patterns in game (García et al., 2020). Given the substantial physiological demands associated with the repeated execution of these high-intensity actions, energy supply in basketball depends on the coordinated contribution of both anaerobic and aerobic metabolic pathways. Accordingly, sprinting capacity, COD performance, and intermittent aerobic endurance are widely recognized as core components of basketball-specific physical fitness and key determinants of competitive performance (Ferioli et al., 2020).

Previous research has shown that basketball players are required to perform a considerable volume of short-distance sprinting and COD actions over the course of a game. Game analyses indicate that players typically cover a total distance of approximately 4.5–5 km per game and perform a high-intensity sprint approximately every 21 s, amounting to more than 105 such efforts within a single game, each lasting roughly 2–6 s (Figueira et al., 2022). In addition, players have been reported to perform between 550 and 1,000 directional changes per game, effectively modifying movement speed or direction every 2 s (Papla et al., 2022; Stojanović et al., 2018). Notably, COD actions generally involve rapid transitions between acceleration and deceleration and are closely intertwined with sprinting performance. Consequently, the efficient execution of sprinting and COD movements is not only essential for offensive and defensive transitions, spatial positioning, and contest situations in basketball, but also exerts a direct influence on overall athletic performance. Considering these competitive demands, the identification of effective training strategies to improve sprinting ability, COD performance, and intermittent aerobic endurance is of substantial practical and scientific significance.

Sprint-oriented training has been extensively employed in team sports such as basketball, soccer, and rugby, owing to its close correspondence with the physiological and mechanical demands of competition (Figueira et al., 2021; Rey et al., 2024). Previous studies have demonstrated that sprint-based training can effectively enhance acceleration capacity, sprint performance, COD performance, and the capacity to repeatedly perform high-intensity efforts in team-sport athletes (Aschendorf et al., 2019; Figueira et al., 2021; Padulo et al., 2015; Rey et al., 2024; Xu et al., 2024). However, the adaptive responses to such training are highly contingent upon the specific structure of the training program and are shaped by both established training principles (e.g., specificity and progression) and key loading parameters, including training intensity, recovery duration, and session frequency (Castagna et al., 2008; Figueira et al., 2021; Haugen et al., 2019; Nicholson et al., 2021; Xu et al., 2024). Existing research has investigated several important variables in sprint training, including alternative sprint training formats, linear versus COD sprint modalities, and different methods of session organization (Attene et al., 2015; Marzouki et al., 2021; Nicholson et al., 2021; Padulo et al., 2015). Nevertheless, sprint distance, one of the key prescription variables in sprint training, has received comparatively limited scholarly attention, particularly in team-sport contexts and, more specifically, within basketball-specific conditioning.

Although previous studies have demonstrated that sprint distance influences acceleration mechanics, maximal running velocity, metabolic stress, and neuromuscular adaptations (Haugen et al., 2019), sprint distance has rarely been examined as an independent programming variable. Instead, it has typically been manipulated concurrently with other training variables, such as total running volume, work-to-rest ratio, training intensity, or sprint frequency (Figueira et al., 2021; Padulo et al., 2015; Rey et al., 2024). Consequently, it remains difficult to isolate the specific contribution of sprint distance to training adaptations, as the observed effects may be confounded by differences in the overall training stimulus. This limitation restricts both the mechanistic understanding of sprint-distance-specific adaptations and the ability of coaches to prescribe sprint-training programs based on evidence regarding sprint distance itself.

From a basketball-specific perspective, most sprint actions performed during competition are short in distance (e.g., 5–10 m), typically occurring during explosive first-step drives, re-acceleration following defensive movements, and short-space positional contests. At the same time, longer sprint efforts (e.g., 20–30 m) also play a meaningful role during fast breaks, defensive recovery runs, and transitional phases of play (Ben Abdelkrim et al., 2010). Accordingly, from the standpoint of training specificity, sprint training performed over different distances may elicit distinct physical adaptations. From a mechanistic perspective, sprint distance is expected to influence the neuromuscular, biomechanical, and metabolic demands imposed during training. Short-distance sprints are performed predominantly within the acceleration phase and require rapid horizontal force production, explosive neuromuscular activation, and acceleration-specific movement coordination, which may preferentially enhance early acceleration performance (Morin et al., 2011). In contrast, longer sprint distances extend into the maximal-velocity phase, requiring sustained force production at higher running speeds, greater eccentric braking before deceleration, and increased glycolytic and cardiovascular demands (Girard et al., 2011; Singh et al., 2025). These characteristics may contribute to improvements in COD performance through enhanced braking and force reorientation, while also promoting intermittent running capacity. Therefore, manipulating sprint distance may differentially influence basketball-specific physical adaptations despite matched external training volume.

Although previous studies have examined the effects of various sprint-training configurations on athletic performance, the existing evidence has been derived predominantly from other team sports, particularly soccer (Rey et al., 2024), while corresponding research in basketball populations remains relatively limited. More importantly, basketball-related studies to date have largely focused on repeated-sprint training (Lin et al., 2026), sprint interval training (Fang and Jiang, 2024), or COD sprint protocols (Brini et al., 2020), with insufficient attention devoted to the discrete training adaptations attributable to sprint distance per se. Moreover, the movement demands of basketball differ substantially from those of soccer and other field-based team sports (Stojanović et al., 2018). Basketball is characterized by a higher frequency of short accelerations, rapid decelerations, multidirectional transitions, and repeated high-intensity efforts performed within a relatively confined playing area (García et al., 2020). Consequently, evidence derived from other sports cannot be directly extrapolated to basketball conditioning. At present, direct comparative evidence remains limited regarding whether sprint training performed over different distances differentially affects multidimensional basketball-specific fitness attributes, including sprint performance, COD performance, and intermittent aerobic endurance.

Thus, the present study aimed to compare the effects of short-distance sprint training (SST) and long-distance sprint training (LST) on sprint performance, COD performance, and intermittent aerobic endurance in collegiate basketball players while matching total external training volume. By controlling external training volume, this study sought to isolate sprint distance as the primary programming variable and determine whether different sprint distances elicit distinct basketball-specific physical adaptations. We hypothesized that both training modalities would improve basketball-specific physical performance. Furthermore, based on the principle of training specificity, we hypothesized that SST would elicit greater improvements in short-distance acceleration performance, whereas LST would produce greater improvements in longer-distance sprint performance, COD performance, and intermittent aerobic endurance.

## Materials and methods

### Participants

**Figure 1:**
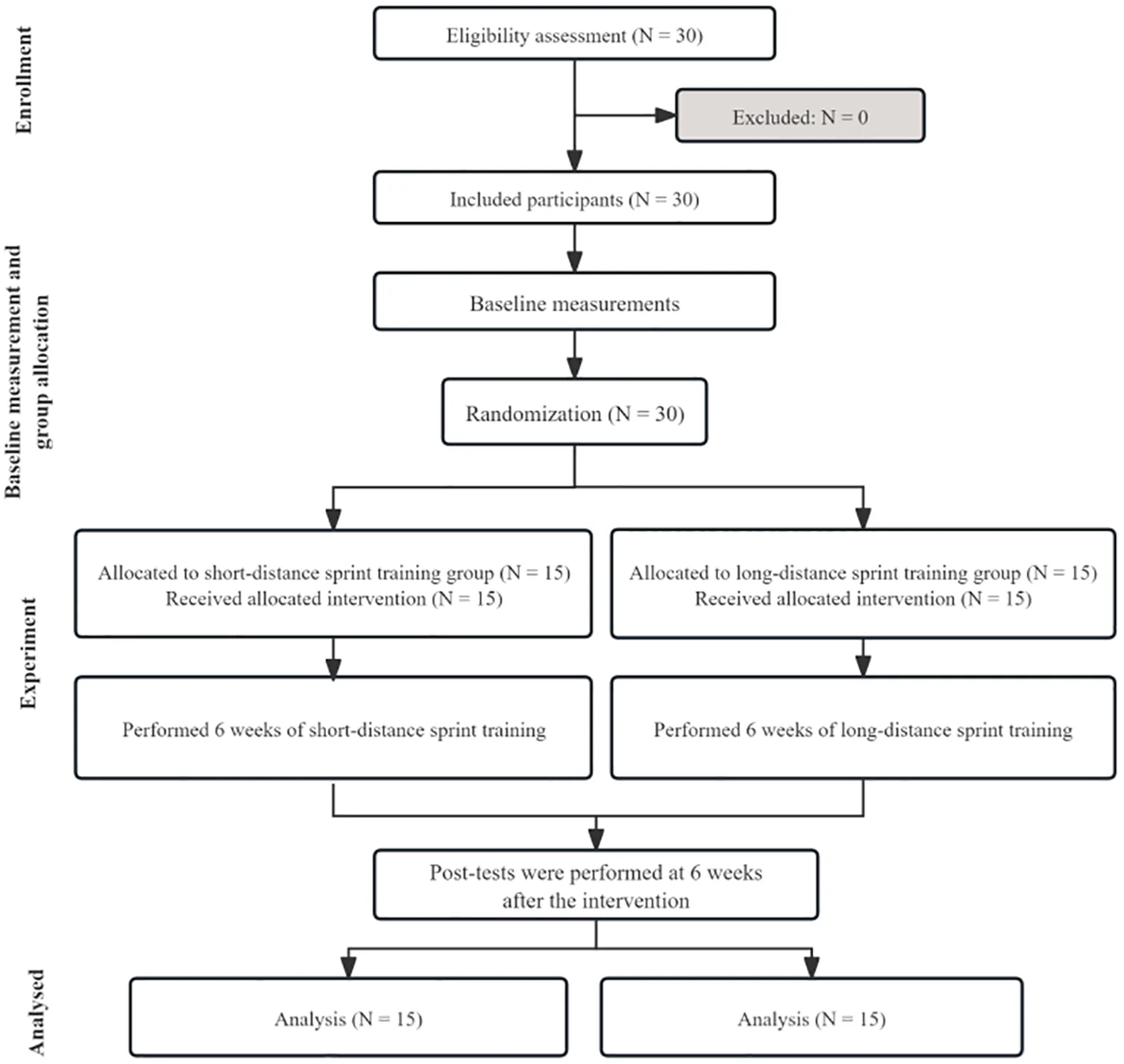
Study flow diagram of participant enrollment, randomization and interventions.

A total of 30 male basketball athletes from the basketball specialization program at Beijing Sport University were recruited for this study. Following baseline assessments, participants were stratified according to playing position (guards, forwards, and centers) and randomly allocated to either the SST (n = 15) or LST (n = 15) group using a computerized randomization application. The randomization sequence was generated by an independent researcher who was not involved in participant recruitment, intervention delivery, outcome assessment, or statistical analysis. Allocation was concealed until completion of all baseline measurements. Outcome assessors remained blinded to group allocation throughout the testing procedures. Sample size estimation was performed using G*Power (3.1.9.2; Heinrich-Heine-University Düsseldorf, Germany) under the F-tests family (ANOVA: Repeated measures, between factors). Based on the intervention effects reported in a previous sprint-training study (Rey et al., 2024), the expected effect size was set at f = 0.50, with an α level of 0.05 and a statistical power (1 − β) of 0.80. Assuming two groups and two repeated measurements (pre and post), the required sample size was estimated to be 26 participants. To account for potential attrition, 30 participants were ultimately enrolled. **Figure 1** illustrates the enrollment, allocation, and attrition of participants. Eligibility criteria included: (1) a minimum of three years of systematic basketball training, with prior experience in both school-level and provincial-level competitions; (2) absence of severe musculoskeletal injury or surgery within the preceding six months; and (3) no known cardiovascular disease, metabolic disorder, or other medical condition contraindicating high-intensity exercise. Exclusion criteria comprised: (1) engagement in regular sprint training within the previous six months; and (2) participation in any additional structured physical training outside the prescribed sprint-training intervention during the study period. Participants were permitted to continue their habitual basketball training throughout the intervention, which was maintained consistently between groups. All participants provided written informed consent after receiving comprehensive information regarding the study objectives, procedures, and potential risks. The study protocol was approved by the Ethics Committee of Beijing Sport University (No. 2025561H) and was conducted in accordance with the Declaration of Helsinki. Furthermore, the trial was registered with the Chinese Clinical Trial Registry (ChiCTR2600122073). Baseline participant characteristics are presented in **Table 1**.

**Table 1:** Basic characteristics of participants.

Variable	SST (n = 15)	LST (n = 15)	*P* value
Age (years)	22.00 ± 2.17	22.27 ± 1.91	0.567
Height (m)	1.82 ± 0.06	1.83 ± 0.06	0.704
Body mass (kg)	81.05 ± 11.55	80.82 ± 10.89	0.707
Body fat (%)	15.81 ± 6.18	17.71 ± 5.11	0.764
Training years (yrs)	5.87 ± 2.90	6.33 ± 1.80	0.064

SST, short-distance sprint training; LST, long-distance sprint training.

### Design and procedures

This study employed a randomized controlled trial design to compare the effects of SST and LST on sprint performance, COD performance, and intermittent aerobic endurance in collegiate basketball athletes. This study was reported in accordance with the CONSORT guidelines. All participants completed a 6-week sprint training intervention, performed twice weekly. In addition to the experimental intervention, all participants maintained their usual schedule of two regular basketball training sessions per week as well as their standard academic commitments, but were instructed to refrain from any additional structured sprint training throughout the study period to minimize potential confounding effects from external training exposure. Before and after the intervention, all participants completed the following performance assessments over a 2-day testing period: 1) 5 m sprint, 2) 10 m sprint, 3) 20 m sprint, 4) 30 m sprint, 5) 505 test, 6) T-test, and 7) Yo-Yo Intermittent Recovery Test Level 1 (Yo-Yo IR1). All tests were performed following a standardized warm-up protocol. The warm-up lasted approximately 20 min and consisted of low-intensity jogging, dynamic stretching, and sport-specific activation exercises (e.g., short sprints and COD drills), with the aim of ensuring an appropriate level of neuromuscular activation and readiness for performance testing. Throughout the 6-week intervention period, participants’ rating of perceived exertion (RPE) was recorded immediately after each training session using the Borg 6–20 scale (Borg, 1998). As participants completed two training sessions per week, the RPE values from the two sessions were averaged to obtain a weekly mean RPE for subsequent analysis of changes in perceived exertion over the intervention period. In addition, all participants completed a standardized familiarization session prior to the commencement of formal testing and training, during which they were familiarized with the testing procedures and provided with opportunities to practice the required movements. To control for external influences, participants were instructed to avoid alcohol, caffeine, and other stimulants that might affect performance for at least 24 h before each testing session, and to obtain no less than 8 h of sleep the night before testing. Upon arrival at each testing session, participants verbally confirmed to the investigators that they had adhered to these pre-test requirements. Furthermore, all pre- and post-intervention assessments were conducted at the same time of day, in the same indoor facility, and by the same team of investigators, to minimize the influence of environmental and procedural variability on the study outcomes.

### Training protocol

All training sessions were conducted on a standard indoor wooden basketball court at the indoor basketball facility of Beijing Sport University. Environmental conditions were maintained as consistently as possible throughout the intervention, with an indoor temperature of approximately 26–28 °C. Participants wore the same pair of their habitual basketball training shoes throughout the intervention. All testing and training sessions were supervised by a basketball coach and a certified strength and conditioning coach holding a National Strength and Conditioning Association certification, to ensure that all participants performed the prescribed training tasks in accordance with the study requirements and with maximal perceived effort. Both coaches also provided standardized verbal encouragement throughout each sprint session to ensure maximal sprint effort during every repetition. The formal training intervention commenced 1 week after baseline testing. The intervention lasted 6 weeks, with participants completing two training sessions per week (12 sessions in total), scheduled on Tuesdays and Saturdays. Each training session lasted approximately 40–50 min. Before each training session, participants completed a standardized 20 min warm-up consisting of light jogging, dynamic mobility exercises (e.g., walking lunges, leg swings, and high-knee drills), and progressive sprint drills (e.g., high-knee running, butt kicks, acceleration runs). To maximize the quality of sprint execution and minimize the potential confounding effects of accumulated fatigue, all sprint training sessions were performed prior to regular basketball-specific practice. The detailed training structure and loading parameters are presented in **Table 2**. Both groups completed a sprint training intervention with matched total training volume, with the only difference being the distance of each sprint repetition. Specifically, participants in the SST group performed 15 m sprints, whereas those in the LST group performed 30 m sprints. For every repetition, participants were instructed to sprint maximally from the starting line and continue running through the designated finish line without decelerating prematurely. Following each sprint, participants decelerated within an additional 8–10 m beyond the finish line before walking back to the starting position during the prescribed recovery period. The recovery interval was set at 15 s for the SST group and 30 s for the LST group, respectively, to maintain a similar work-to-rest ratio between groups and to allow participants to perform repeated near-maximal sprint efforts throughout the intervention period. To monitor physical activity performed beyond the prescribed intervention, all participants were required to complete an online physical activity questionnaire every Sunday evening, documenting any additional weekly physical activity undertaken outside the study (Sallis et al., 1985). Training attendance was recorded at every session, and all participants completed 100% of the prescribed training sessions, with no missed training sessions throughout the intervention period.

**Table 2:** Overview of the sprint training protocols for the short-distance sprint training and long-distance sprint training groups.

Week	Group	Distance (m)	Recovery (s)	Repetition	Distance/session (m)	Distance/week (m)
1	SST	15	15	12	180	360
	LST	30	30	6	180	360
2	SST	15	15	12	180	360
	LST	30	30	6	180	360
3	SST	15	15	14	210	420
	LST	30	30	7	210	420
4	SST	15	15	14	210	420
	LST	30	30	7	210	420
5	SST	15	15	16	240	480
	LST	30	30	8	240	480
6	SST	15	15	16	240	480
	LST	30	30	8	240	480

SST, short-distance sprint training; LST, long-distance sprint training.

### Outcome measures

To comprehensively evaluate the effects of sprint training on physical performance, assessments of linear sprint speed, COD performance, and aerobic capacity were performed. Because COD performance is a multifactorial ability, three complementary assessments (505 test, T-test, and COD deficit (CODD)) were included to evaluate braking and re-acceleration capacity, multidirectional agility, and COD efficiency independent of linear sprint speed, respectively.

### Sprint performance

Sprint performance was assessed using 5 m, 10 m, 20 m, and 30 m linear sprint tests. Sprint times were recorded automatically using an electronic timing system (Smartspeed, Fusion Sport Inc., Australia) with a precision of 0.001 s. All sprint assessments were conducted over a 40 m straight-line section of an indoor basketball court. Five pairs of infrared timing gates were positioned at the start line and at 5 m, 10 m, 20 m, and 30 m, respectively, to capture split times across each distance. All participants performed the test using a standing start, with the lead foot positioned 0.5 m behind the initial timing gate. Participants were permitted to select their preferred lead foot, but were required to maintain the same starting stance across all trials to minimize measurement variability. Each participant completed three maximal sprint trials, separated by 2–3 min of passive recovery. Standardized verbal encouragement was provided throughout testing. The fastest time recorded across the three trials was retained for subsequent statistical analysis (Rey et al., 2024).

### COD performance

#### T test

The T-test was used to assess multidirectional COD performance. Test time was recorded automatically using an electronic timing system (Smartspeed, Fusion Sport, Australia). At the start of the test, participants adopted a standing start position, with the lead foot placed 0.5 m behind the timing gate. Upon the start command, participants sprinted forward 9.14 m to touch the first cone. They then shuffled 4.57 m to the left to touch the second cone, followed by a 9.14 m lateral shuffle to the right to touch the third cone. Participants subsequently shuffled 4.57 m back to the left to return to the first cone, and finally backpedaled 9.14 m through the finish timing gate to complete the test. Each participant completed three valid trials, with 3 min of passive recovery between trials. The best performance was retained for subsequent statistical analysis (Cuenca-Garcia et al., 2022).

#### 505 test

The 505 test was used to assess participants’ 180° turning and re-acceleration performance. Test time was recorded automatically using an electronic timing system (Smartspeed, Fusion Sport, Australia). At the start of the test, participants began from a standing position 0.5 m behind the first timing gate and performed a maximal 15 m linear sprint. Additional timing gates were positioned at the 5 m and 10 m marks along the sprint path to record split times. Upon reaching the 15 m turning line, participants were required to plant one foot on or beyond the line, execute a rapid 180° turn, and immediately sprint back 5 m. The test was considered complete once the participant passed back through the timing gate positioned at the 10 m mark. To minimize the potential influence of directional asymmetry, all participants performed the test using their preferred turning direction, which was kept consistent between pre- and post-intervention assessments. Each participant completed two valid trials, with 3 min of passive recovery between trials. The best trial was retained for subsequent statistical analysis (Fernandez-Fernandez et al., 2023). In addition, the CODD was calculated to provide a more isolated indicator of COD efficiency. CODD was calculated as follows: CODD = 5-0–5 time − 10 m sprint time. A lower CODD value generally indicates less time loss during the COD task relative to linear sprinting, thereby reflecting greater COD efficiency (Dos’Santos et al., 2019; Wen et al., 2018).

#### YO-YO intermittent recovery test

Yo-Yo IR1 was performed according to the procedures suggested by Selmi et al (Selmi et al., 2023). During the test, participants performed repeated 20 m shuttle runs between two markers positioned 20 m apart, following a pre-recorded audio signal that dictated running pace. After each completed 2 × 20 m shuttle bout, participants were provided with 10 s of active recovery. As the test progressed, the interval between successive audio signals gradually decreased, thereby requiring participants to increase their running speed to maintain the prescribed pace. At the beginning of each shuttle, participants were required to have at least one foot in contact with the starting line and to reach the corresponding turning line or return line before the subsequent audio signal. Failure to maintain the required pace was recorded as a missed attempt. The test was terminated when a participant accumulated two consecutive failures to reach the line in time. The total distance completed was recorded and used as the outcome measure for subsequent statistical analysis.

### Statistical analysis

All statistical analyses were conducted using IBM SPSS Statistics 28 (SPSS Inc., Chicago, IL, USA). Data are presented as means ± standard deviations (SDs). Normality and homogeneity of variance were assessed using the Shapiro–Wilk and Levene’s tests, respectively, and all variables satisfied these assumptions. Baseline differences between groups were examined using one-way analysis of variance (ANOVA). Training effects were examined using a 2 (group: SST vs. LST) × 2 (time: pre vs. post) repeated-measures ANOVA for all performance variables, whereas RPE was analyzed using a 2 (group) × 6 (time: weeks 1–6) repeated-measures ANOVA. Sphericity was assessed using Mauchly’s test, and Greenhouse–Geisser corrections were applied when necessary. Significant interaction or main effects were followed by Bonferroni-adjusted pairwise comparisons. Effect sizes for ANOVA were expressed as partial eta squared (η^2p^), interpreted as trivial (≤ 0.01), small (> 0.01–0.06), moderate (> 0.06–0.14), and large (> 0.14). Within-group effect sizes were calculated using Cohen’s d and interpreted as trivial (0–0.20), small (> 0.20–0.60), moderate (> 0.60–1.20), large (> 1.20–2.00), and very large (> 2.00). In addition to p-values and effect sizes, 95% confidence intervals (95% CI) are reported where appropriate to facilitate estimation-based interpretation of the observed effects. All participants completed both the intervention and post-intervention assessments; therefore, no missing data were present and no imputation procedures were required. Statistical significance was set at *p* < 0.05.

## Results

At baseline, no statistically significant between-group differences were observed between the SST and LST groups in height, body mass, age, body fat percentage, or training years (*p* > 0.05). No training-related adverse events were reported throughout the intervention, suggesting that both training protocols were safe and practically feasible.

### Sprint performance

**Figure 2:**
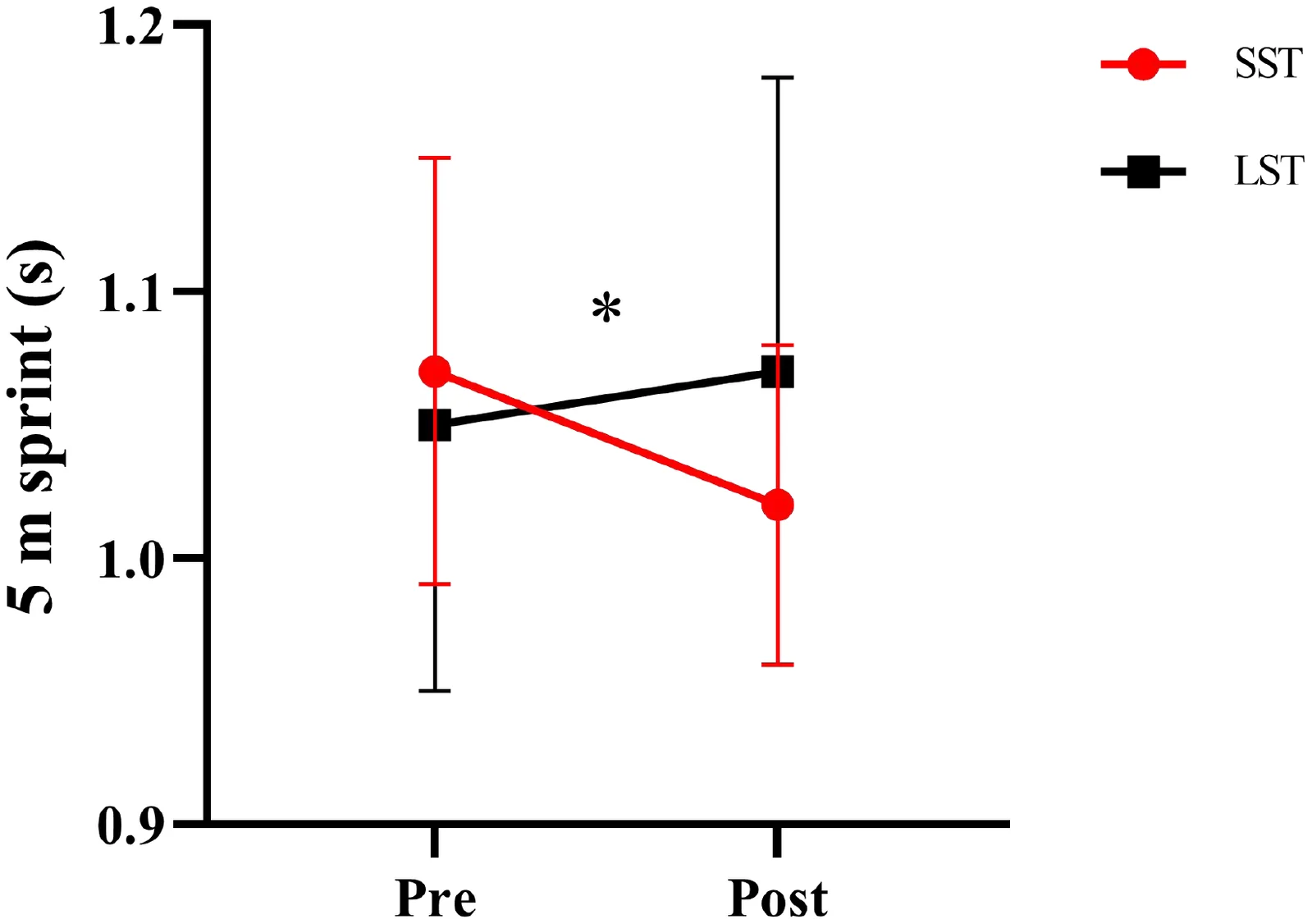
Comparison of 5 m sprint performance between the SST group and the LST group before and after the intervention. SST, short-distance sprint training; LST, long-distance sprint training; * Significant interaction between the time and the group.

The two-way repeated-measures ANOVA revealed a significant time × group interaction only for 5 m sprint performance (*F* = 7.99, *p* = 0.022, η²_p_ = 0.222). No significant interaction effects were observed for 10 m (*F* = 2.346, *p* = 0.137, η²_p_ = 0.077), 20 m (*F* = 0.289, *p* = 0.595, η²_p_ = 0.01), or 30 m sprint performance (*F* = 1.629, *p* = 0.212, η²_p_ = 0.055). *Post hoc* analysis further demonstrated that the SST group exhibited a significantly greater improvement in 5 m sprint performance than the LST group (*p* = 0.007; see **Figure 2**). Regarding main effects, significant effects of time were observed for 10 m (*F* = 12.217, *p* = 0.002, η²_p_ = 0.304), 20 m (*F* = 4.482, *p* = 0.043, η²_p_ = 0.138), and 30 m sprint performance (*F* = 21.155, *p* < 0.001, η²_p_ = 0.43), whereas no significant group main effects were detected (*p* > 0.05). Subsequent *post hoc* comparisons for the time effect indicated that 10 m sprint performance improved significantly following the intervention in the SST group (*p* = 0.001), while 20 m sprint performance in the LST group showed a trend toward improvement (*p* = 0.071). For 30 m sprint performance, both the SST (*p* = 0.026) and LST groups (*p* < 0.001) demonstrated significant post-intervention improvements. Detailed within-group changes are presented in **Table 3**.

**Table 3:** Changes in sprint, change-of-direction, and aerobic endurance performance before and after 6 weeks of training in the SST and LST groups.

Variables	SST					LST				
	Pre	Post	Mean difference (95% CI)	Δ (%)	ES	Pre	Post	Mean difference (95% CI)	Δ (%)	ES
5 m (s)	1.07 ± 0.08	1.02 ± 0.06	-0.05 (-0.08--0.01)	-4.67	0.71	1.05 ± 0.10	1.07 ± 0.11	0.02 (-0.02-0.05)	+1.90	0.19
10 m (s)	1.82 ± 0.08	1.77 ± 0.09	-0.05 (-0.08--0.02)	-2.75	0.59	1.81 ± 0.14	1.79 ± 0.13	-0.02 (-0.05-0.01)	-1.10	0.15
20 m (s)	3.08 ± 0.15	3.05 ± 0.14	-0.03 (-0.07-0.02)	-0.97	0.21	3.10 ± 0.22	3.06 ± 0.20	-0.04 (-0.09-0.00)	-1.29	0.19
30 m (s)	4.31 ± 0.22	4.25 ± 0.22	-0.06 (-0.12--0.01)	-1.39	0.27	4.40 ± 0.35	4.29 ± 0.30	-0.01 (-0.17--0.06)	-2.50	0.34
505 test (s)	2.52 ± 0.13	2.43 ± 0.10	-0.10 (-0.15--0.04)	-3.57	0.78	2.57 ± 0.15	2.47 ± 0.16	-0.1 (-0.08--0.01)	-3.89	0.64
T test (s)	9.09 ± 0.45	8.90 ± 0.43	-0.19 (-0.32--0.06)	-2.09	0.43	9.46 ± 0.74	9.13 ± 0.66	-0.32 (-0.46--0.19)	-3.49	0.47
CODD (s)	0.71 ± 0.14	0.66 ± 0.11	-0.05 (-0.11-0.08)	-7.04	0.40	0.77 ± 0.13	0.69 ± 0.12	-0.08 (-0.15--0.02)	-10.39	0.64
Yo-Yo IR1 (m)	762.67 ± 289.02	896.00 ± 333.91	133.33 (83.23-183.44)	+17.48	0.43	826.67 ± 343.32	1008.00 ± 388.17	181.33 (131.23-231.44)	+21.95	0.50

CODD, change of direction deficit; Yo-Yo IR1, Yo-Yo Intermittent Recovery Test Level 1; SST, short-distance sprint training; LST, long-distance sprint training; 95% CI, 95% confidence intervals; ES, Cohen’s d.

### COD performance

The two-way repeated-measures ANOVA showed no significant time × group interactions for the 505 test (*F* = 0.005, *p* = 0.946, η²_p_ < 0.001), T-test (*F* = 2.247, *p* = 0.145, η²_p_ = 0.074), or CODD (*F* = 0.553, *p* = 0.463, η²_p_ = 0.019). However, significant main effects of time were found for the 505 test (*F* = 25.728, *p* < 0.001, η²_p_ = 0.479), T-test (*F* = 31.847, *p* < 0.001, η²_p_ = 0.532), and CODD (*F* = 8.297, *p* = 0.008, η²_p_ = 0.229), whereas no significant group main effects were identified (*p* > 0.05). *Post hoc* analysis of the time effect indicated that both the SST group (*p* = 0.001 and *p* = 0.007, respectively) and the LST group (*p* = 0.001 and *p* < 0.001, respectively) improved significantly in the 505 test and T-test following the intervention. In addition, a significant improvement in CODD was observed only in the LST group (*p* = 0.016). Detailed within-group changes are presented in **Table 3**.

### Intermittent aerobic endurance

For the Yo-Yo IR1 test, the two-way repeated-measures ANOVA revealed no significant time × group interaction (*F* = 1.925, *p* = 0.176, η²_p_ = 0.064) and no significant group main effect (*F* = 0.511, *p* = 0.481, η²_p_ = 0.018). However, a significant main effect of time was observed (*F* = 82.74, *p* < 0.001, η²_p_ = 0.747). *Post hoc* comparisons indicated that intermittent aerobic endurance improved significantly from pre- to post-intervention in both the SST group (*p* < 0.001) and the LST group (*p* < 0.001). Detailed within-group changes are presented in **Table 3**.

### RPE

The two-way repeated-measures ANOVA demonstrated a significant main effect of time for RPE (*F* = 5.192, *p* = 0.002, η²_p_ = 0.156). However, neither the time × group interaction (*F* = 1.021, *p* = 0.39, η²_p_ = 0.035) nor the group main effect (*F* = 0.126, *p* = 0.725, η²_p_ = 0.004) reached statistical significance. Further *post hoc* analysis of the time effect showed that none of the pairwise comparisons between time points reached statistical significance. *Post hoc* analysis further showed that, within the SST group, RPE in week 5 was significantly higher than that in weeks 3 (*p* = 0.05), 4 (*p* = 0.023), and 6 (*p* < 0.001), whereas within the LST group, RPE in week 5 was significantly higher than that in week 6 (*p* < 0.001).

## Discussion

The present study aimed to compare the effects of SST and LST on sprint performance, COD performance, and intermittent aerobic endurance in collegiate basketball players. The principal findings indicate that, following the 6-week intervention, both sprint training modalities effectively improved sprint performance, COD performance, and intermittent aerobic endurance, thereby broadly supporting the study hypothesis. A significant group × time interaction was observed only for 5 m sprint performance, indicating greater improvements following SST than LST. In contrast, no significant between-group differences were found for the remaining sprint, COD, intermittent aerobic endurance, or RPE outcomes, although both groups demonstrated significant improvements over time. Although descriptively greater improvements were observed following LST for several performance measures, these differences did not reach statistical significance. Collectively, these findings further support sprint training as an effective method for enhancing sprinting, COD performance, and intermittent aerobic endurance in basketball players, while also suggesting that sprint distance may partially influence the specificity of physical adaptations relevant to basketball conditioning.

Sprint speed is widely regarded as one of the key physical determinants of basketball performance, particularly in contexts requiring rapid offensive and defensive transitions, explosive first-step movements, and dynamic off-ball actions. Previous research has indicated that sprint ability may distinguish basketball players across competitive levels (Ben Abdelkrim et al., 2010; Goniotaki et al., 2026). Consequently, training strategies aimed at enhancing sprint performance are of considerable practical relevance within basketball conditioning programs. In the present study, under matched training volume conditions, a significant group × time interaction was observed only for 5 m sprint performance, indicating that SST was more effective than LST in improving early acceleration. For the remaining sprint distances, both training protocols improved sprint performance over time, with no significant between-group differences. Although the overall pattern of adaptations was generally consistent with the principle of training specificity, statistical evidence supporting differential adaptations was limited to the 5 m sprint (Rey et al., 2024). Sprint performance is generally characterized by three interrelated phases: initial acceleration, maximal velocity attainment, and speed maintenance (von Lieres Und Wilkau et al., 2020). SST may preferentially target explosive initiation and acceleration over the first few steps, which are frequently required in basketball-specific scenarios such as short-distance penetration, closeout recovery, and rapid acceleration following a steal to initiate a fast break. In contrast, LST may be more conducive to developing the ability to sustain higher running velocities, which is particularly relevant during full-court fast breaks, extended defensive recovery efforts, and transition-based sprinting actions. This distinction may help explain the descriptive pattern of adaptations observed in the present study. Specifically, SST tended to show numerically greater improvements in shorter sprint distances, whereas LST tended to show numerically greater improvements over longer sprint distances. However, these descriptive trends should be interpreted cautiously because statistically significant between-group differences were observed only for the 5 m sprint. From a mechanistic perspective, sprint training may enhance sprint performance through improvements in neuromuscular recruitment and neural adaptation (Henz and Schöllhorn, 2016). Repeated exposure to high-intensity sprint stimuli may increase the ability to store elastic energy during the eccentric phase and subsequently release greater mechanical energy during the concentric phase. Enhanced utilization of the stretch-shortening cycle may therefore provide a progressively stronger training stimulus over time, ultimately contributing to improvements in sprint performance (Arede et al., 2021).

An additional finding deserving consideration is that the LST group did not demonstrate an improvement in 5 m sprint performance. This may reflect the greater emphasis of longer sprint training on maximal-velocity running rather than rapid force generation during the initial acceleration phase. Because adaptations are generally specific to the velocity and force demands imposed during training, LST may provide a relatively weaker stimulus for improving first-step acceleration. Alternatively, this observation may also be attributable to normal biological variability and the relatively small sample size. Therefore, this finding should be interpreted cautiously and warrants confirmation in future studies. Although the observed improvements in sprint performance were relatively small (approximately 1–3%), they may still be practically meaningful in basketball, where game outcomes often depend on rapid first-step acceleration and transition speed. Even modest improvements in sprint performance may enhance an athlete’s ability to gain or recover positional advantage during decisive game situations.

To the best of our knowledge, this is the first study to directly compare the effects of different sprint distances within a basketball-specific population. As such, direct comparisons with previous basketball-based studies remain limited. Although the present findings are broadly consistent with those reported by (Rey et al., 2024), several methodological differences should be considered when interpreting the comparison. Rey et al. investigated adolescent soccer players using 20 m and 40 m sprint protocols, whereas the present study examined collegiate basketball players using shorter sprint distances (15 m vs. 30 m). These differences in participant characteristics, sport-specific demands, and sprint-training prescriptions may partly explain why only the 5 m sprint demonstrated a significant between-group difference in the present study.

COD ability plays a critical role in basketball performance, particularly in defensive shuffling, offensive separation, and transitional play (Miller et al., 2006). In general, previous studies have suggested that meaningful improvements in COD ability typically require training stimuli with a high degree of movement specificity, rather than being derived solely from linear sprint-based interventions (Maio Alves et al., 2010; Sáez de Villarreal et al., 2021). Nevertheless, the present findings demonstrated that both SST and LST improved 505 test performance, T-test performance, and CODD over time. Although descriptively greater improvements were observed following LST, no significant group × time interactions were detected for any COD outcome. Therefore, these descriptive differences should be interpreted cautiously and do not provide conclusive evidence that LST is superior to SST for improving COD performance. These results suggest that, even in the absence of highly specific COD training stimuli, sprint training may still exert a positive transfer effect on COD ability. The improvements in COD performance observed in the present study are likely multifactorial, with the most plausible mechanisms involving enhanced lower-limb force production, improved linear sprint ability, and augmented neural adaptations. First, high-quality COD performance depends on an athlete’s ability to execute rapid braking, directional reorientation, and re-acceleration within an extremely short ground contact period, which requires substantial rate of force development and explosive power capacity (Miller et al., 2006). To perform these actions effectively, athletes must generate efficient eccentric braking through the lower-limb extensor musculature during the deceleration phase to rapidly reduce momentum and lower the center of mass, followed by rapid concentric force production to propel the body in the new direction (Sheppard and Young, 2006; Spiteri et al., 2015). Previous evidence has shown that faster athletes in COD tasks tend to exhibit greater ground reaction forces and shorter contact times during the penultimate and final foot contacts, thereby enabling more efficient deceleration and re-acceleration mechanics (Dos'Santos et al., 2020). In addition, Andrés et al (Baena-Raya et al., 2022). emphasized that a high level of horizontal force production and its effective application are critical for maximizing acceleration capacity, which may likewise contribute to enhanced COD performance and reduced CODD. Compared with repeated 15 m accelerations, repeated 30 m sprints expose athletes to higher running velocities and a longer maximal-speed phase, thereby increasing horizontal force application as well as eccentric braking demands before deceleration. Efficient COD performance requires athletes not only to generate high propulsive forces during re-acceleration but also to rapidly absorb braking forces and effectively redirect momentum. Therefore, repeated exposure to higher-speed running may enhance the neuromuscular qualities required for braking control and force re-orientation, potentially contributing to the descriptively greater improvements observed following LST (Morin et al., 2011; Sheppard and Young, 2006; Singh et al., 2025). Improvements in linear sprint ability may also help explain the observed enhancement in COD performance. Specifically, enhanced sprint ability may contribute not only to greater approach velocity prior to the directional change but also to improved exit velocity following the turn (Vanrenterghem et al., 2012). Previous studies have consistently reported significant associations between linear sprint speed and COD performance (Delextrat et al., 2015; Mikołajec et al., 2022; Scanlan et al., 2014). Taken together, these findings support the notion that improvements in sprint performance may partially underpin the enhancement in COD outcomes observed following both sprint training interventions. Finally, neural adaptations may represent another important mechanism contributing to the improvement in COD ability. Sprint training may enhance neuromuscular function by increasing motor unit recruitment, firing frequency, and intermuscular coordination, thereby improving the athlete’s capacity to generate force rapidly under high-speed and high-force conditions (Wang et al., 2022). Such adaptations may not only facilitate improvements in linear sprint performance but may also enhance braking, force absorption, and re-acceleration capacities during directional changes. Future studies should incorporate biomechanical and neuromuscular assessments to directly verify the mechanisms underlying improvements in COD performance following sprint training.

Aerobic capacity is of particular importance in basketball, as it underpins the repeated execution of high-intensity actions such as sprinting, decelerating, and changing direction during both offensive and defensive phases of play (Aschendorf et al., 2019). A relatively high level of intermittent aerobic endurance is therefore considered an essential physiological foundation for sustaining high-intensity basketball-specific performance. In the present study, the total distance covered during the Yo-Yo IR1 test increased significantly in both the SST and LST groups following the 6-week intervention, indicating that both sprint training modalities were effective in improving intermittent aerobic endurance in collegiate basketball players. Both groups demonstrated significant improvements in Yo-Yo IR1 performance over time, whereas no significant group × time interaction was observed. Although the LST group demonstrated a descriptively greater improvement, this difference should be interpreted cautiously because it was not statistically significant. The observed enhancement in intermittent aerobic endurance may be attributable to both central and peripheral adaptations induced by sprint training (Song et al., 2023). Compared with SST, repeated 30 m sprints require athletes to sustain high-intensity running for a longer duration during each repetition, thereby increasing glycolytic energy contribution, cardiovascular demand, and the cumulative time spent near maximal running velocity (Buchheit and Laursen, 2013). These greater metabolic demands may provide a stronger stimulus for adaptations in oxidative capacity, mitochondrial enzyme activity, and fatigue resistance, all of which contribute to intermittent running performance assessed by the Yo-Yo IR1 test (Macpherson and Weston, 2015). From a central perspective, repeated sprint-based exercise may improve oxygen delivery capacity, whereas from a peripheral perspective, it may enhance the skeletal muscle’s capacity to extract and utilize oxygen more efficiently (Bailey et al., 2009; Thurlow et al., 2024). Importantly, both SST and LST share the hallmark characteristics of high-intensity intermittent exercise and may therefore provide a sufficient stimulus to elicit meaningful improvements in intermittent aerobic endurance (Nimphius et al., 2016). However, this interpretation remains speculative and should be confirmed by future studies incorporating direct physiological measurements. Such adaptations are highly relevant to basketball performance, which depends not merely on the ability to perform a single sprint, but rather on the capacity to repeatedly execute high-intensity efforts throughout the course of an entire game. The present findings are also broadly consistent with previous evidence demonstrating improvements in aerobic performance following sprint-based training interventions (Fang and Jiang, 2024; Macpherson and Weston, 2015). Future studies incorporating physiological measurements such as heart rate responses, blood lactate concentration, oxygen uptake, and muscle oxidative capacity are warranted to clarify the mechanisms through which sprint distance influences intermittent running performance.

The present study also monitored RPE throughout the 6-week intervention to characterize the internal load associated with the two sprint training protocols. The results showed that overall RPE values were comparable between groups and followed a broadly similar pattern over time, suggesting that SST and LST imposed a relatively similar perceptual training load. These findings indicate that, when total training volume is matched, differences in sprint distance may not substantially alter the overall subjective training stimulus experienced by the athletes.

Despite the valuable insights provided by the present study, several limitations should be acknowledged. First, the absence of a non-sprint control group limits causal inference regarding the independent effects of sprint training beyond regular basketball practice. Therefore, the present findings should be interpreted as comparative adaptations between the SST and LST protocols rather than evidence for the absolute efficacy of sprint training. Second, although an *a priori* sample size calculation indicated that the recruited sample was sufficient, the relatively small sample size may have limited the statistical power to detect smaller between-group differences. Moreover, although baseline training experience did not differ significantly between groups (p = 0.064), its potential influence on individual responsiveness cannot be completely excluded. Future studies with larger samples and more balanced baseline characteristics are warranted. Third, the trial was registered retrospectively after participant recruitment had commenced. Although the study protocol and predefined outcome measures remained unchanged following registration, prospective registration would further strengthen methodological transparency. Fourth, although total sprint distance and the work-to-rest ratio were matched, the physiological equivalence of the training stimuli was not objectively verified. Future studies should incorporate objective measures of both internal and external training load (e.g., heart rate, blood lactate concentration, Global Positioning System-derived movement metrics, and sprint velocity) to better characterize the physiological and physical demands associated with different sprint-distance protocols. Fifth, the intervention lasted only six weeks without follow-up assessments and included only male collegiate basketball players. Therefore, caution should be exercised when generalizing these findings to other populations or inferring long-term training adaptations. Finally, although the present study demonstrated differential performance adaptations between sprint-distance protocols, it did not directly investigate the neuromuscular and biomechanical mechanisms underlying these responses. Future studies incorporating electromyography, force–velocity profiling, muscle architecture, and tendon mechanical properties may provide further insight into the mechanisms responsible for these adaptations.

## Conclusion

The present study demonstrates that both SST and LST are effective in improving sprint performance, COD performance, and intermittent aerobic endurance in male collegiate basketball players. A significant advantage of SST was observed only for 5 m sprint performance. Although SST and LST showed different descriptive patterns of adaptation across several performance outcomes, no significant between-group differences were observed for the remaining sprint, COD, or intermittent aerobic endurance measures. These findings that sprint distance may be considered as one of several programming variables when designing sprint training for male collegiate basketball players.

### Practical applications

Our findings suggest that sprint distance may be considered as one of several programming variables when designing sprint training for male collegiate basketball players. From a practical perspective, SST may be particularly applicable when the training objective is to enhance repeated short accelerations commonly required during first-step penetration, close-out defense, ball-pressure situations, and rapid reactions in confined spaces. In contrast, LST may be incorporated when greater emphasis is placed on repeated full-court transition sprinting, defensive recovery runs, and longer offensive–defensive transition actions. Furthermore, coaches may consider incorporating different sprint distances according to specific training objectives, training phase, positional demands, and individual athlete characteristics.

## Data Availability

The original contributions presented in the study are included in the article/**Supplementary Material**. Further inquiries can be directed to the corresponding author.

## References

[B1] Arede J. Poureghbali S. Freitas T. Fernandes J. Schöllhorn W. I. Leite N. (2021). The effect of differential repeated sprint training on physical performance in female basketball players: A pilot study. Int. J. Environ. Res. Public Health 18, 12616. doi: 10.3390/ijerph182312616 34886342 PMC8656732

[B2] Aschendorf P. F. Zinner C. Delextrat A. Engelmeyer E. Mester J. (2019). Effects of basketball-specific high-intensity interval training on aerobic performance and physical capacities in youth female basketball players. Phys. Sportsmed 47, 65–70. doi: 10.1080/00913847.2018.1520054 30193074

[B3] Attene G. Laffaye G. Chaouachi A. Pizzolato F. Migliaccio G. M. Padulo J. (2015). Repeated sprint ability in young basketball players: One vs. two changes of direction (part 2). J. Sports Sci. 33, 1553–1563. doi: 10.1080/02640414.2014.996182 25574803

[B4] Baena-Raya A. Jiménez-Reyes P. Romea E. S. Soriano-Maldonado A. Rodríguez-Pérez M. A. (2022). Gender-specific association of the sprint mechanical properties with change of direction performance in basketball. J. Strength Cond Res. 36, 2868–2874. doi: 10.1519/jsc.0000000000003974 33555826

[B5] Bailey S. J. Wilkerson D. P. Dimenna F. J. Jones A. M. (2009). Influence of repeated sprint training on pulmonary O2 uptake and muscle deoxygenation kinetics in humans. J. Appl. Physiol. (1985) 106, 1875–1887. doi: 10.1152/japplphysiol.00144.2009 19342439

[B6] Ben Abdelkrim N. Chaouachi A. Chamari K. Chtara M. Castagna C. (2010). Positional role and competitive-level differences in elite-level men's basketball players. J. Strength Cond Res. 24, 1346–1355. doi: 10.1519/JSC.0b013e3181cf7510 20393355

[B7] Borg G. (1998). Borg's Perceived Exertion and Pain Scales (Champaign: Human kinetics).

[B8] Brini S. Ben Abderrahman A. Boullosa D. Hackney A. C. Zagatto A. M. Castagna C. . (2020). Effects of a 12-week change-of-direction sprints training program on selected physical and physiological parameters in professional basketball male players. Int. J. Environ. Res. Public Health 17, 8214. doi: 10.3390/ijerph17218214 33172136 PMC7664328

[B9] Buchheit M. Laursen P. B. (2013). High-intensity interval training, solutions to the programming puzzle. Part II: Anaerobic energy, neuromuscular load and practical applications. Sports Med. 43, 927–954. doi: 10.1007/s40279-013-0066-5 23832851

[B10] Castagna C. Abt G. Manzi V. Annino G. Padua E. D'Ottavio S. (2008). Effect of recovery mode on repeated sprint ability in young basketball players. J. Strength Cond Res. 22, 923–929. doi: 10.1519/JSC.0b013e31816a4281 18438220

[B11] Cuenca-Garcia M. Marin-Jimenez N. Perez-Bey A. Sánchez-Oliva D. Camiletti-Moiron D. Alvarez-Gallardo I. C. . (2022). Correction to: Reliability of field-based fitness tests in adults: A systematic review. Sports Med. 52, 1981–1982. doi: 10.1007/s40279-022-01654-7 35064915

[B12] Delextrat A. Grosgeorge B. Bieuzen F. (2015). Determinants of performance in a new test of planned agility for young elite basketball players. Int. J. Sports Physiol. Perform. 10, 160–165. doi: 10.1123/ijspp.2014-0097 24956606

[B13] Dos'Santos T. McBurnie A. Thomas C. Comfort P. Jones P. A. (2020). Biomechanical determinants of the modified and traditional 505 change of direction speed test. J. Strength Cond Res. 34, 1285–1296. doi: 10.1519/jsc.0000000000003439 31868815

[B14] Dos’Santos T. Thomas C. Jones P. A. Comfort P. (2019). Assessing asymmetries in change of direction speed performance: Application of change of direction deficit. J. Strength Cond Res. 33, 2953–2961. doi: 10.1519/jsc.0000000000002438 29373434

[B15] Fang K. Jiang H. (2024). Gender-specific effects of short sprint interval training on aerobic and anaerobic capacities in basketball players: A randomized controlled trial. J. Sports Sci. Med. 23, 8–16. doi: 10.52082/jssm.2024.8 38455442 PMC10915601

[B16] Ferioli D. Schelling X. Bosio A. La Torre A. Rucco D. Rampinini E. (2020). Match activities in basketball games: Comparison between different competitive levels. J. Strength Cond Res. 34, 172–182. doi: 10.1519/jsc.0000000000003039 30741861

[B17] Fernandez-Fernandez J. Canós-Portalés J. Martínez-Gallego R. Corbi F. Baiget E. (2023). Effects of different maturity status on change of direction performance of youth tennis players. Biol. Sport 40, 867–876. doi: 10.5114/biolsport.2023.121324 37398953 PMC10286620

[B18] Figueira B. Gonçalves B. Abade E. Paulauskas R. Masiulis N. Kamarauskas P. . (2021). Repeated sprint ability in elite basketball players: The effects of 10 × 30 m vs. 20 × 15 m exercise protocols on physiological variables and sprint performance. J. Hum. Kinet. 77, 181–189. doi: 10.2478/hukin-2020-0048 34168703 PMC8008292

[B19] Figueira B. Mateus N. Esteves P. Dadelienė R. Paulauskas R. (2022). Physiological responses and technical-tactical performance of youth basketball players: A brief comparison between 3x3 and 5x5 basketball. J. Sports Sci. Med. 21, 332–340. doi: 10.52082/jssm.2022.332 35719227 PMC9157511

[B20] García F. Vázquez-Guerrero J. Castellano J. Casals M. Schelling X. (2020). Differences in physical demands between game quarters and playing positions on professional basketball players during official competition. J. Sports Sci. Med. 19, 256–263. PMC7196749 32390718

[B21] Girard O. Mendez-Villanueva A. Bishop D. (2011). Repeated-sprint ability - part I: Factors contributing to fatigue. Sports Med. 41, 673–694. doi: 10.2165/11590550-000000000-00000 21780851

[B22] Goniotaki A. Bourdas D. I. Travlos A. K. Bakirtzoglou P. Theos A. Zacharakis E. (2026). Physical and performance profiles differentiate competitive levels in U-18 basketball players. Sports (Basel) 14, 27. doi: 10.3390/sports14010027 41590969 PMC12845602

[B23] Haugen T. Seiler S. Sandbakk Ø. Tønnessen E. (2019). The training and development of elite sprint performance: An integration of scientific and best practice literature. Sports Med. - Open 5, 44. doi: 10.1186/s40798-019-0221-0 31754845 PMC6872694

[B24] Henz D. Schöllhorn W. I. (2016). Differential training facilitates early consolidation in motor learning. Front. Behav. Neurosci. 10, 199. doi: 10.3389/fnbeh.2016.00199 27818627 PMC5073148

[B25] Lin Y. Zhang W. Zhao L. Yang Y. (2026). Effect of change-of-direction vs. linear repeated sprint training on physical performance in female college basketball players. Sci. Rep. 16, 10939. doi: 10.1038/s41598-026-45810-9 41896656 PMC13039901

[B26] Macpherson T. W. Weston M. (2015). The effect of low-volume sprint interval training on the development and subsequent maintenance of aerobic fitness in soccer players. Int. J. Sports Physiol. Perform. 10, 332–338. doi: 10.1123/ijspp.2014-0075 25203817

[B27] Maio Alves J. M. Rebelo A. N. Abrantes C. Sampaio J. (2010). Short-term effects of complex and contrast training in soccer players' vertical jump, sprint, and agility abilities. J. Strength Cond Res. 24, 936–941. doi: 10.1519/JSC.0b013e3181c7c5fd 20300035

[B28] Marzouki H. Ouergui I. Doua N. Gmada N. Bouassida A. Bouhlel E. (2021). Effects of 1 vs. 2 sessions per week of equal-volume sprint training on explosive, high-intensity and endurance-intensive performances in young soccer players. Biol. Sport 38, 175–183. doi: 10.5114/biolsport.2020.97675 34079162 PMC8139356

[B29] Mikołajec K. Gabryś T. Gryko K. Prończuk M. Krzysztofik M. Trybek G. . (2022). Relationship among the change of direction ability, sprinting, jumping performance, aerobic power and anaerobic speed reserve: A cross-sectional study in elite 3x3 basketball players. J. Hum. Kinet. 85, 105–113. doi: 10.2478/hukin-2022-0114 36643835 PMC9808805

[B30] Miller M. G. Herniman J. J. Ricard M. D. Cheatham C. C. Michael T. J. (2006). The effects of a 6-week plyometric training program on agility. J. Sports Sci. Med. 5, 459–465. PMC3842147 24353464

[B31] Morin J. B. Edouard P. Samozino P. (2011). Technical ability of force application as a determinant factor of sprint performance. Med. Sci. Sports Exerc 43, 1680–1688. doi: 10.1249/MSS.0b013e318216ea37 21364480

[B32] Nicholson B. Dinsdale A. Jones B. Till K. (2021). The training of short distance sprint performance in football code athletes: A systematic review and meta-analysis. Sports Med. 51, 1179–1207. doi: 10.1007/s40279-020-01372-y 33245512 PMC8124057

[B33] Nimphius S. Callaghan S. J. Spiteri T. Lockie R. G. (2016). Change of direction deficit: A more isolated measure of change of direction performance than total 505 time. J. Strength Cond Res. 30, 3024–3032. doi: 10.1519/jsc.0000000000001421 26982972

[B34] Padulo J. Laffaye G. Haddad M. Chaouachi A. Attene G. Migliaccio G. M. . (2015). Repeated sprint ability in young basketball players: One vs. two changes of direction (part 1). J. Sports Sci. 33, 1480–1492. doi: 10.1080/02640414.2014.992936 25530125

[B35] Papla M. Perenc D. Zając A. Maszczyk A. Krzysztofik M. (2022). Contribution of strength, speed and power characteristics to change of direction performance in male basketball players 12, 8484. doi: 10.3390/app12178484

[B36] Rey E. Carrera S. Padrón-Cabo A. Costa P. B. (2024). Effectiveness of short vs. long-distance sprint training on sprinting and agility performance in young soccer players. Biol. Sport 41, 87–93. doi: 10.5114/biolsport.2024.127384 38188118 PMC10765434

[B37] Sáez de Villarreal E. Molina J. G. de Castro-Maqueda G. Gutiérrez-Manzanedo J. V. (2021). Effects of plyometric, strength and change of direction training on high-school basketball player's physical fitness. J. Hum. Kinet. 78, 175–186. doi: 10.2478/hukin-2021-0036 34025875 PMC8120961

[B38] Sallis J. F. Haskell W. L. Wood P. D. Fortmann S. P. Rogers T. Blair S. N. . (1985). Physical activity assessment methodology in the Five-City Project. Am. J. Epidemiol. 121, 91–106. doi: 10.1093/oxfordjournals.aje.a113987 3964995

[B39] Scanlan A. Humphries B. Tucker P. S. Dalbo V. (2014). The influence of physical and cognitive factors on reactive agility performance in men basketball players. J. Sports Sci. 32, 367–374. doi: 10.1080/02640414.2013.825730 24015713

[B40] Selmi W. Hammami R. Kasmi S. Sehli S. Rebai H. Duncan M. . (2023). Effects of aerobic and speed training versus active control on repeated sprint ability and measures of self-confidence and anxiety in highly trained male soccer players. Sports Med. - Open 9, 63. doi: 10.1186/s40798-023-00619-y 37498373 PMC10374512

[B41] Sheppard J. M. Young W. B. (2006). Agility literature review: Classifications, training and testing. J. Sports Sci. 24, 919–932. doi: 10.1080/02640410500457109 16882626

[B42] Singh U. Leicht A. S. Connor J. D. Brice S. M. Alves A. Doma K. (2025). Biomechanical determinants of change of direction performance: A systematic review. Sports Med. 55, 2207–2224. doi: 10.1007/s40279-025-02278-3 40668491 PMC12476310

[B43] Song T. Jilikeha Deng Y. (2023). Physiological and biochemical adaptations to a sport-specific sprint interval training in male basketball athletes. J. Sports Sci. Med. 22, 605–613. doi: 10.52082/jssm.2023.605 38045752 PMC10690500

[B44] Spiteri T. Newton R. U. Binetti M. Hart N. H. Sheppard J. M. Nimphius S. (2015). Mechanical determinants of faster change of direction and agility performance in female basketball athletes. J. Strength Cond Res. 29, 2205–2214. doi: 10.1519/jsc.0000000000000876 25734779

[B45] Stojanović E. Stojiljković N. Scanlan A. T. Dalbo V. J. Berkelmans D. M. Milanović Z. (2018). The activity demands and physiological responses encountered during basketball match-play: A systematic review. Sports Med. 48, 111–135. doi: 10.1007/s40279-017-0794-z 29039018

[B46] Thurlow F. Huynh M. Townshend A. McLaren S. J. James L. P. Taylor J. M. . (2024). The effects of repeated-sprint training on physical fitness and physiological adaptation in athletes: A systematic review and meta-analysis. Sports Med. 54, 953–974. doi: 10.1007/s40279-023-01959-1 38041768

[B47] Vanrenterghem J. Venables E. Pataky T. Robinson M. A. (2012). The effect of running speed on knee mechanical loading in females during side cutting. J. Biomech. 45, 2444–2449. doi: 10.1016/j.jbiomech.2012.06.029 22835648

[B48] von Lieres Und Wilkau H. C. Irwin G. Bezodis N. E. Simpson S. Bezodis I. N. (2020). Phase analysis in maximal sprinting: An investigation of step-to-step technical changes between the initial acceleration, transition and maximal velocity phases. Sports Biomech. 19, 141–156. doi: 10.1080/14763141.2018.1473479 29972337

[B49] Wang Z. H. Pan R. C. Huang M. R. Wang D. (2022). Effects of integrative neuromuscular training combined with regular tennis training program on sprint and change of direction of children. Front. Physiol. 13, 831248. doi: 10.3389/fphys.2022.831248 35222092 PMC8867084

[B50] Wen N. Dalbo V. J. Burgos B. Pyne D. B. Scanlan A. T. (2018). Power testing in basketball: Current practice and future recommendations. J. Strength Cond Res. 32, 2677–2691. doi: 10.1519/jsc.0000000000002459 29401204

[B51] Xu H. Song J. Li G. Wang H. (2024). Optimal prescription for superior outcomes: A comparative analysis of inter-individual variability in adaptations to small-sided games and short sprint interval training in young basketball players. J. Sports Sci. Med. 23, 305–316. doi: 10.52082/jssm.2024.305 38841633 PMC11149073

